# Analysis of postoperative complications 5 years after osteosynthesis of patella fractures—a retrospective, multicenter cohort study

**DOI:** 10.1007/s00068-024-02503-0

**Published:** 2024-04-03

**Authors:** Markus T. Berninger, Alexander Korthaus, Lena Eggeling, Elmar Herbst, Mirjam V. Neumann-Langen, Christoph Domnick, Kai Fehske, Stefan Barzen, Clemens Kösters, Johannes Zellner, Michael J. Raschke, Karl-Heinz Frosch, Reinhard Hoffmann, Matthias Krause

**Affiliations:** 1 Department of Trauma and Orthopaedic Surgery, University Medical Center Hamburg-Eppendorf, Hamburg, Germany; 2 Department of Trauma, Orthopaedic Surgery, and Sports Traumatology, BG Hospital Hamburg, Hamburg, Germany; 3 Department of Trauma, Hand and Reconstructive Surgery, University Hospital Münster, Münster, Germany; 4 Department of Orthopaedic and Trauma Surgery, Klinikum Konstanz, Constance, Germany; 5 Department of Trauma, Hand and Orthopedic Surgery, Euregio-Hospital, Nordhorn, Germany; 6 Department of Orthopaedic and Trauma Surgery, Johanniter Waldkrankenhaus, Bonn, Germany; 7 Department of Orthopaedic Trauma, Hand, Plastic and Reconstructive Surgery, University Hospital Würzburg, Würzburg, Germany; 8 Department of Trauma and Orthopaedic Surgery, Berufsgenossenschaftliche Unfallklinik Frankfurt Am Main, Frankfurt Am Main, Germany; 9 Department of Orthopedics and Trauma Surgery, Maria and Josef Hospital, Greven, Germany; 10 Sporthopaedicum Regensburg, Regensburg, Germany

**Keywords:** Patella fracture, Complication, Tension band wiring, Locking plate osteosynthesis, Screw osteosynthesis, Revision surgery

## Abstract

**Purpose:**

The study aims to investigate the influence of patient- and fracture-specific factors on the occurrence of complications after osteosynthesis of patella fractures and to compare knee joint function, activity, and subjective pain levels after a regular postoperative course and after complications in the medium term.

**Methods:**

This retrospective, multicenter cohort study examined patients who received surgery for patella fracture at level 1 trauma centers between 2013 and 2018. Patient demographics and fracture-specific variables were evaluated. Final follow-up assessments included patient-reported pain scores (NRS), subjective activity and knee function scores (Tegner Activity Scale, Lysholm score, IKDC score), complications, and revisions.

**Results:**

A total of 243 patients with a mean follow-up of 63.4 ± 21.3 months were included. Among them, 66.9% of patients underwent tension band wiring (TBW), 19.0% received locking plate osteosynthesis (LPO), and 14.1% underwent screw osteosynthesis (SO). A total of 38 patients (15.6%) experienced complications (TBW: 16.7%; LPO: 15.2%; SO: 11.8%). Implant-related complications of *atraumatic fragment dislocation* and *material insufficiency/dislocation*, accounted for 50% of all complications, were significantly more common after TBW than LPO (*p* = 0.015). No patient-specific factor was identified as a general cause for increased complications. Overall, particularly following complications such as *limited range of motion* or *traumatic refracture*, functional knee scores were significantly lower and pain levels were significantly higher at the final follow-up when a complication occurred. Implant-related complications, however, achieved functional scores comparable to a regular postoperative course without complications after revision surgery.

**Conclusion:**

The present study demonstrated that implant-related complications occurred significantly more often after TBW compared to LPO. The complication rates were similar in all groups.

## Introduction

Patella fractures are relatively rare accounting for approximately 1% of all fractures [1, 2]. An accurate anatomical reconstruction of the articular surface plays a crucial role in surgical treatment and leads to the best functional results [3]. This requires a thorough understanding of the morphologic characteristics of the fracture and an osteosynthesis tailored to the fracture. Tension band wiring (TBW), locking plate osteosynthesis (LPO), and (cannulated) screw osteosynthesis (SO) are established surgical treatment options [4]. Numerous biomechanical and clinical studies have described the expected postoperative function and rates of complications for different osteosynthesis methods [5–9]. However, functional results are often unsatisfactory, especially after TBW, with 30–70% of patients reporting functional limitations [3, 10]. This may be explained by the relatively high rates of postoperative complications. After TBW, rates of complications of up to 56% have been described in the literature primarily including material insufficiency or dislocation, fracture displacement, secondary fragment dislocation, and infection/soft tissue irritation [3, 6, 11]. The rates of complications in the few studies of LPO (such as loss of reduction due to dislocated pole fragments, reactive prepatellar bursitis, infection) are significantly lower, but also not negligible with rates of up to 15% [12, 13].

There are numerous causes for complications after patella fracture osteosynthesis requiring surgical revision. The primary objective of this retrospective, multicenter cohort study was to evaluate the influence of patient- and fracture-specific factors on the occurrence of complications. Second, differences in relation to knee joint function, activity level, and subjective pain will be analyzed after a normal postoperative course and after a complication in the medium term.

## Patients and methods

### Study group

**Fig. 1:**
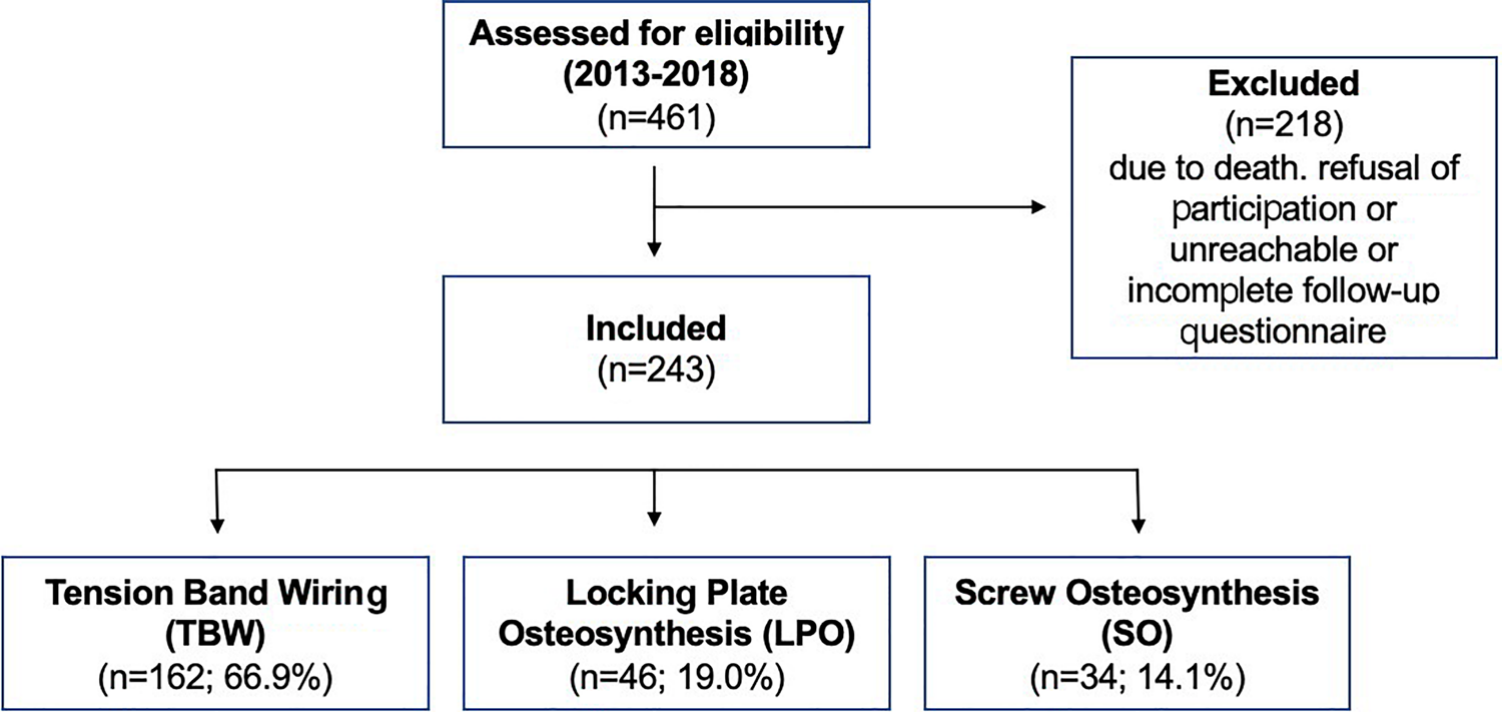
Flow chart of recruitment process. FiberWire sutures alone were used exclusively in one patient

This retrospective, multicenter cohort study analyzed patients who underwent surgery for a patella fracture at level-one trauma centers between January 2013 and December 2018. The study included patients aged 17 years or older with a minimum follow-up of 24 months. Patients without informed consent for study participation or incomplete questionnaires for function and pain level scoring were excluded. All surgical procedures consisted of open reduction and internal fixation using either TBW, anterior LPO, or SO (Fig. 1).

### Outcome measures

Data for outcome measures were obtained at final follow-up through questionnaires sent to the patients, which included functional outcome and pain score assessments. Patient epidemiological data were evaluated by reviewing each patient’s medical records, surgery protocol, and radiographs. Additional patient-specific factors such as BMI, tobacco and alcohol consumption, and osteoporosis were also analyzed. Osteoporosis assessment was based solely on patient reports; no additional DXA scans or vitamin D level measurements were performed. All fractures were classified utilizing the AO/OTA classification based on the review of preoperative radiographs [14]. Documentation of fracture-specific factors included pole fracture, soft tissue injury, concomitant injuries, and the surgical procedure. Subjective scores for activity and knee function, including Tegner Activity Scale, Lysholm score, and IKDC score, as well as self-reported pain scores in terms of numeric rating scores (NRS) at rest and at motion (0 = no pain; 10 = worst pain) were requested at final follow up. Additionally, the evaluation included complications and revisions. Implant removal was documented and was not considered a complication if conducted at least 6 months postoperatively for common reasons of removing of osteosynthesis material, such as patient distraction by the implant. A restricted range of motion was considered a flexion deficit of < 90° or an extension deficit of > 10°.

The study has been performed in accordance with the ethical standards laid down in the 1964 Declaration of Helsinki and its later amendments. The study protocol was approved by the local ethics committee (2020–10068-BO).

### Statistical analysis

Statistical analysis was carried out using IBM SPSS Statistics version 26.0 (IBM, Armonk, NY) and the software R package stats (version 4.2.2.). Data were presented as mean values ± standard deviation for continuous variables. The data set was split into different subgroups (e.g., with and without complication, different types of complication). A Shapiro–Wilk normality test and Kolmogorov–Smirnov test were performed to determine if the data were normally distributed. Fisher’s exact test was utilized to compare binomial data. When dealing with two independent groups containing parametric and non-parametric data, we applied the independent *T*-test and Mann–Whitney *U* test, respectively. To assess the comparison of two paired groups involving parametric and non-parametric data, we employed the paired *T*-test and Wilcoxon signed-rank test, respectively. In cases where multiple observations were compared, we conducted an ANOVA analysis. A *p* value of < 0.05 was considered statistically significant.

## Results

### Demographics

A total of 243 patients (130 female, 113 male) were included in this study; all patients completed the functional outcome and pain scoring measures at a mean follow-up of 63.4 ± 21.3 months (range 26–110), postoperatively. The mean age of the patients at the time of surgery was 54.8 ± 15.4 years (range: 17–88 years), and the mean BMI was 25.2 ± 4.8 kg/m^2^. As a mechanism of injury, *n* = 104 patients (42.8%) sustained injuries during activities of daily living, *n* = 86 (35.4%) at work, *n* = 32 (13.1%) due to traffic accidents and *n* = 6 (2.5%) during sports, and *n* = 15 (6.2%) for other reasons.

As a primary radiological diagnostic, nearly all patients (93.0%) received a preoperative x-ray of the knee, while 29.6% also received a CT scan instead of or in addition to the x-ray. In 99.6% of patients, a postoperative native radiograph was obtained, with 10.7% receiving postoperative CT scans. According to the AO/OTA classification, we analyzed three cases of 34-type A1 fracture (1.2%), 22 cases of 34-type B fracture (B1: *n* = 12 (4.9%), B2: *n* = 10 (4.1%)), and 218 cases of 34-type C fracture (C1: *n* = 67 (27.6%), C2: *n* = 45 (18.5%), C3: *n* = 106 (43.6%)). Patients with AO 34-type C3-fractures were distributed equally among those under 65 years (43.3%) and those over 65 years (43.7%). The correlation between the AO/OTA classification and preoperative CT scans indicated that CT scans were exclusively performed for C fractures, depending on the severity of the fracture. Specifically, 4.5% of 34-type C1 fractures, 28.9% of 34-type C2 fractures, and 40.6% of 34-type C3 fractures underwent preoperative CT scans. Additionally, 19.8% of all fractures were classified as open fractures according to Gustilo and Anderson’s classification. A total of 17.7% of patients had associated injuries, including additional fractures or soft tissue injuries of the upper or lower extremities.

Surgery was performed on a mean of 3.0 ± 5.2 days (range: 0–28 days) after trauma. The procedures included TBW in 66.9% of patients, LPO in 19.0%, and SO in 14.1%. FiberWire sutures alone were used exclusively in one patient. Supplemental stabilization material was used in 20.3% of patients: 8.0% received a McLaughlin-Cerclage and 12.3% received additional single screws. A total of 76.1% of 34-type C1 fractures, 64.4% of 34-type C2 fractures, and 68.9% of 34-type C3 fractures underwent TBW. LPO was performed in 7.5% of 34-type C1 fractures, 24.5% of 34-type C2 fractures, and 26.4% of 34-type C3 fractures, while SO was used in 14.9% of 34-type C1 fractures, 11.1% of 34-type C2 fractures, and 4.7% of 34-type C3 fractures.

All patients followed a standardized rehabilitation protocol, which involved either partial or full weight bearing (in extension) while wearing a brace that limited flexion to 30° for the initial 2 weeks, 60° for another 2 weeks, and 90° over the following 2 weeks. Implant removal was performed in 122 patients (50.8%) at 11.3 ± 10.7 months postoperatively for various reasons, such as discomfort caused by the implant or patient’s request (TBW: 56,7%, LPO: 45,6%, SO: 23,5%). Removal was not considered a complication if no material insufficiency or dislocation was found.

### Complication analysis

**Fig. 2:**
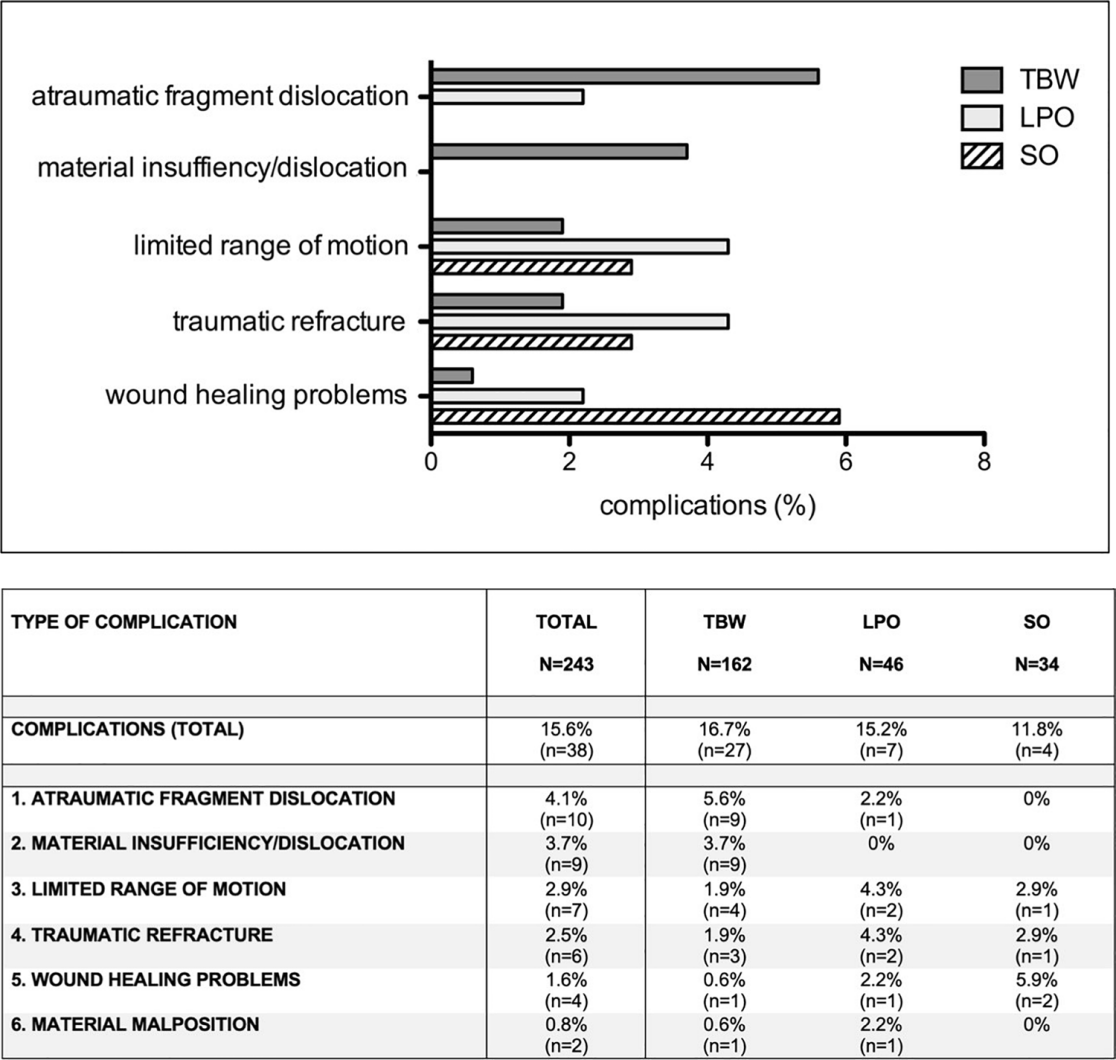
Distribution of types of complications and surgical procedures. TBW, tension band wiring; LPO, locking plate osteosynthesis; SO, screw osteosynthesis

Overall, complications occurred in 38 patients (15.6%). Among these, TBW had the highest rate of complications (16.7%) compared to LPO (15.2%) and SO (11.8%). However, these differences were not statistically significant (*p* = 0.733). The two main causes of complications in this cohort, accounting for 50% of all complications, were *atraumatic fragment dislocation* and *material insufficiency/dislocation*. These complications occurred significantly more common after TBW than LPO (*p* = 0.015) (Fig. 2). In contrast, complications such as *limited range of motion*, *traumatic refracture*, and *wound healing problems* were slightly more common in patients with LPO or SO. Figure 2 provides a detailed distribution of the types of complications and surgical procedures.

In general, patient-specific factors such as age, sex, BMI, regular use of tobacco or alcohol, or osteoporosis were not significant predictors of complications (Table 1). Among fracture-specific factors, distal pole fracture (*p* = 0.801), open fracture (*p* = 0.102), and surgical procedure (*p* = 0.624) did not result in a significantly increased (general) complication rate. However, AO/OTA classification (*p* = 0.020) and concomitant injuries (*p* = 0.015) significantly influenced the occurrence of complications. Complications occurred exclusively in AO 34-type C fractures, resulting in a significantly increased complication rate for AO 34-type C fractures compared to B-type fractures (*p* = 0.033).

**Table 1:**
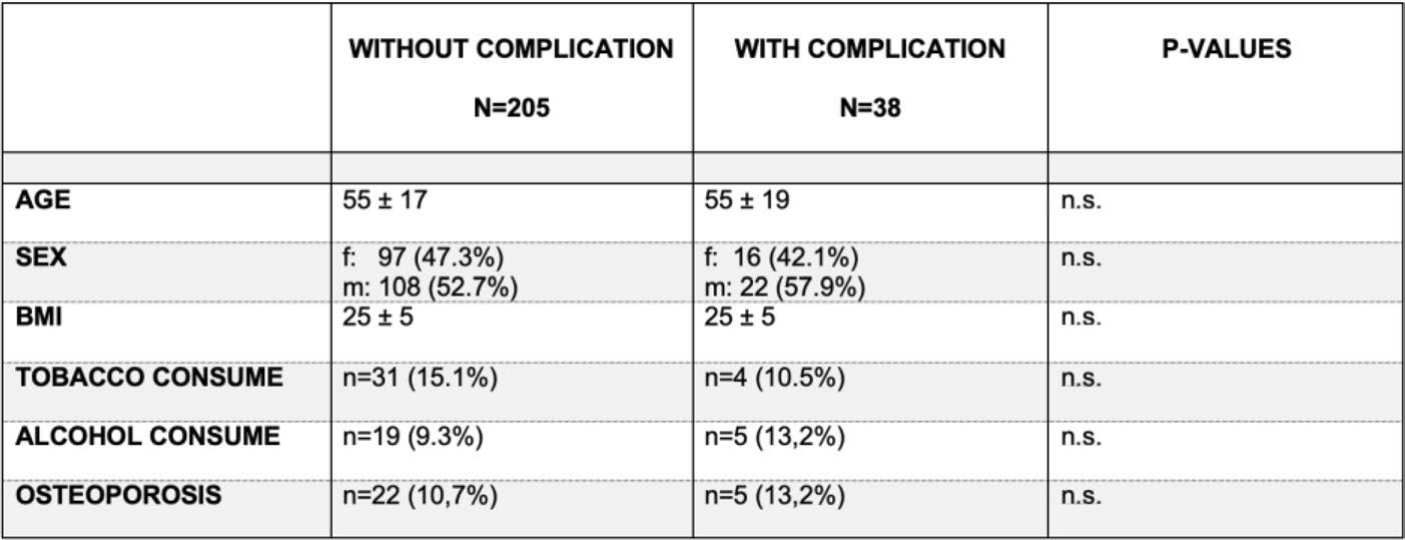
Distribution of patient-specific factors without and with complications*. f*, female;* m*, male; *n.s.*, not significant

However, the analysis of the various types of complications revealed that the occurrence of some of these complications is significantly influenced by patient- and fracture-specific factors, as depicted in Table 2.

**Table 2:**
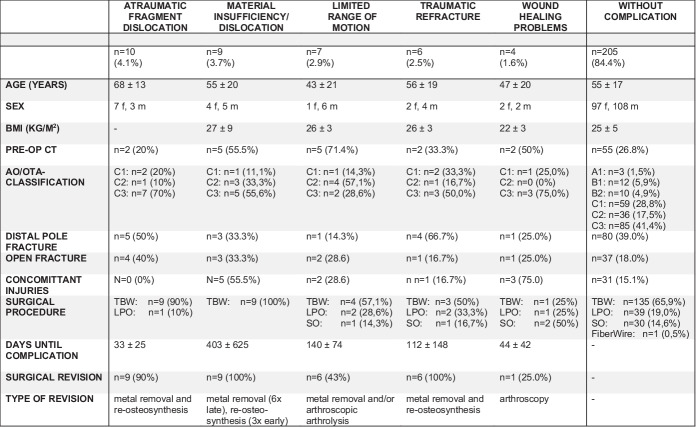
Analysis of the distribution of patient- and fracture-specific factors among different types of complications. *f*, female; *m*, male; *TBW*, tension band wiring; *LPO*, locking plate osteosynthesis; *SO*, screw osteosynthesis

Notably, patients with an *atraumatic fragment dislocation* were significantly older (68 ± 13 years, *p* = 0.012) than patients with other types of complications (50 ± 19 years). Only 20% of patients underwent a preoperative CT scan, but a total of 70% of the reviewed patients had comminuted 34-type C3 fractures. A total of 90% of patients with an *atraumatic fragment dislocation* were initially treated surgically with TBW, and one patient (10%) received a LPO. An *atraumatic fragment dislocation* occurred at an early postoperative stage, around 4–5 weeks after surgery. Almost all complications were revised by implant removal and revision osteosynthesis.

*Material insufficiency/dislocation* was observed exclusively after TBW, which occurred either in the early (≤ 3 weeks) postoperative period (33.3%) or months/years later (66.7%) (mean time of 403 ± 625 days; overall range of 0–1685 days).

In contrast, *limited range of motion* was primarily observed in relatively younger (43 ± 21 years) men, which occurred after 140 ± 47 days and appeared to be independent of the surgical procedure. All patients with *limited range of motion* underwent revision surgery involving implant removal and/or arthroscopic arthrolysis after sufficient bony consolidation of the fracture was achieved.

*Traumatic refracture* mainly occurred in middle-aged patients (56 ± 19 years), independent of the surgical procedure. This complication primarily manifested within the first year (112 ± 148 days, range: 1–393), either early during (< 2.5 months; 66.7%) or late (> 5 months, 33.3%) after osseous consolidation.

*Wound healing problems* occurred relatively early after surgery (44 ± 42 days, range 14–92 days) and could mainly be treated conservatively. There were insufficient cases of *wound healing problems* (*n* = 4) and *material malposition* (*n* = 2) for detailed statistical analysis.

**Fig. 3:**
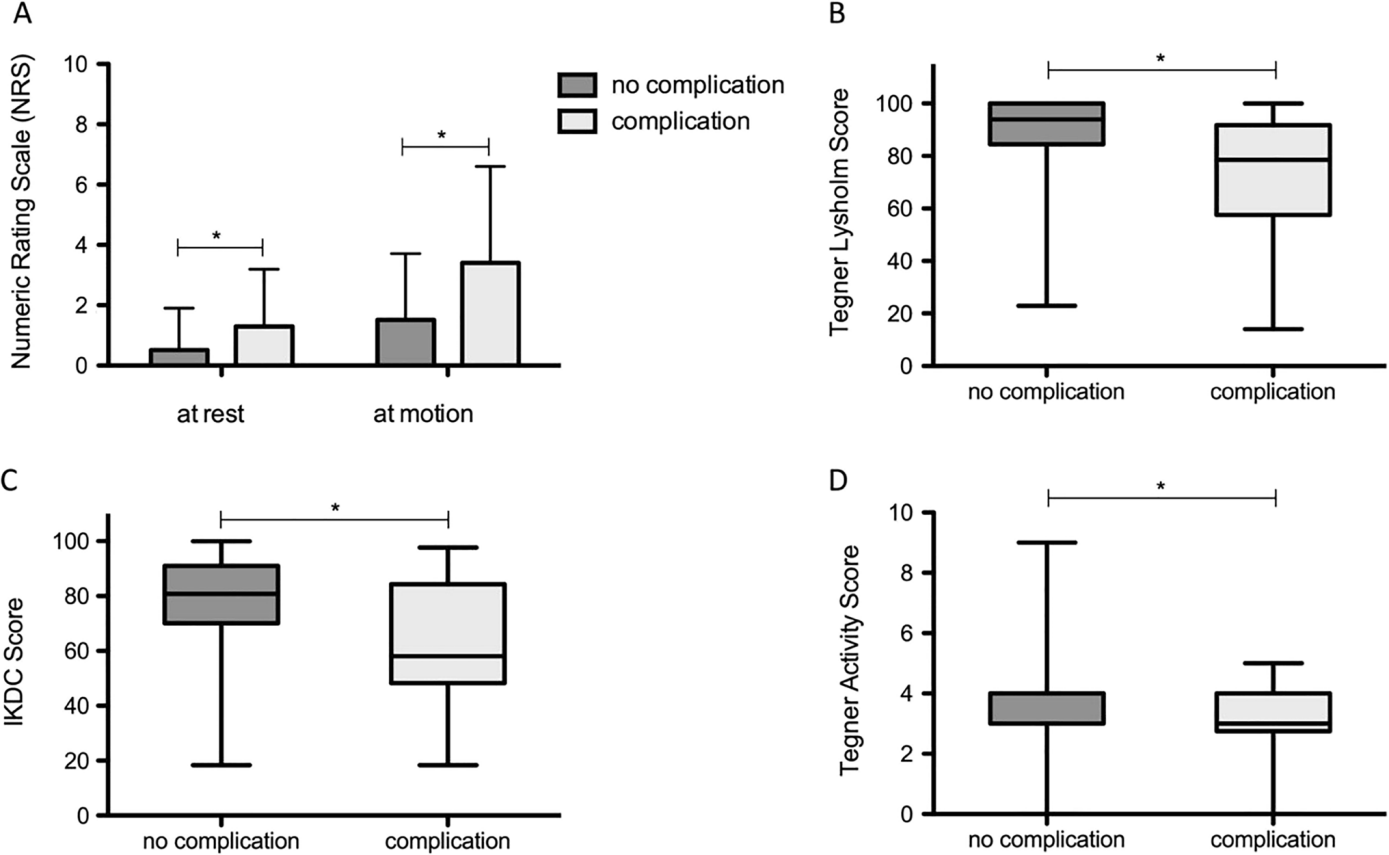
Pain levels (NRS at rest and at motion), functional knee scores (Lysholm and IKDC score), and activity level (Tegner Activity Scale) of patients with and without complications (* *p* < 0.05)

At the final follow-up (63.4 ± 21.3 months after surgery), analysis of pain levels measured by NRS showed significantly higher pain levels at rest after complications (1.3 ± 1.9) compared to patients without complications (0.5 ± 1.4, *p* = 0.0025) (Fig. 3). NRS values at motion were nearly three times higher. In contrast to patients without complications (1.5 ± 2.2, *p* = 0.004), patients with complications had significantly higher pain scores at motion (3.4 ± 3.2). Functional knee scores, including Lysholm score (88.4 ± 15.9 vs. 73.4 ± 20.4, *p* < 0.0001) and IKDC score (78.7 ± 16.8 vs. 62.5 ± 22.1, *p* = 0.0201), as well as the activity level measured by Tegner Activity Scale (3.9 ± 1.3 vs. 3.1 ± 1.3, 0.0021) were also significantly lower after complications.

The analysis of the outcome parameters for the different types of complications revealed that patients with complications of *limited range of motion* or *traumatic refracture* experienced significantly higher pain levels (at rest: *p* = 0.006; at motion: *p* = 0.001) and lower functional scores compared to implant-related complications such as *atraumatic fragment dislocation* and *material insufficiency/dislocation*. Although the final follow-up was 45 ± 13 months after the occurrence of complications and 62 ± 33 months after the initial surgery, patients reported higher pain scores. In addition, implant-related complications had significantly higher functional scores (Lysholm: *p* = 0.001 and IKDC: *p* < 0.001) and activity scores (Tegner: *p* = 0.049) compared to other types of complications (Table 3). Furthermore, no significant differences in functional knee score values were found between patients with *atraumatic fragment dislocation* and *material insufficiency/dislocation* and those without complications (Lysholm: *p* = 0.206; IKDC: *p* = 0.555; Tegner: *p* = 0.224).

**Table 3:**
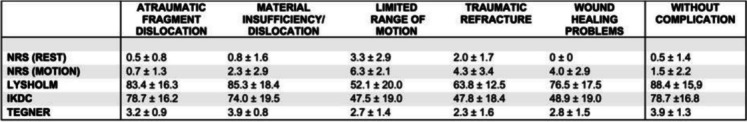
Analysis of the pain and functional outcome scores among different types of complications

## Discussion

The present study demonstrated that implant-related complications of *atraumatic fragment dislocation* and *material insufficiency* accounted for 50% of all complications in the cohort and occurred significantly more often after TBW compared to LPO. No patient-specific factors were found to universally promote complications. However, fracture-dependent factors such as AO/OTA classification and concomitant injuries significantly influenced the occurrence of a complication. Overall, and in particular after complications of *limited range of motion* or *traumatic refracture*, functional knee scores were significantly lower and pain levels were higher up to 5 years after complications. Implant-related complications of *atraumatic fragment dislocation* and *material insufficiency*, however, achieved functional scores after revision surgery that were comparable to a regular postoperative course. These findings underscore the importance of a thorough preoperative evaluation of the fracture and the selection of a fracture-specific osteosynthesis procedure.

Depending on the osteosynthesis method used, patella fractures have a relatively high rate of complications, which is in the literature significantly lower after LPO compared to TBW [12, 13]. Material loosening and dislocation, secondary fracture dislocation, and limited range of motion are the most common complications, resulting in a high rate of revision surgery [3, 11, 15].

In our cohort, patient-specific factors such as age, sex, BMI, regular tobacco or alcohol consumption, and osteoporosis did not significantly affect the occurrence of complications. Notably, age and osteoporosis were found to be complication-independent factors, which is interesting given the increase in complex multi-fragment patella fractures in older patients due to the aging of society [16, 17]. One potential factor contributing to the complexity of fractures is the increased prevalence of osteoporosis in older women, which greatly elevates the risk of multi-fragment fractures resulting from falls caused by low-energy trauma [18, 19]. Although this cohort did not exhibit a direct increase in complications related to osteoporosis, the association remains a valid concern.

On the other hand, Miller et al. demonstrated a correlation between higher failure rates and the use of a TBW with K-wires in elderly patients (> 65 years) [20]. The poor stability of the K-wires of the TBW in osteoporotic bone is a possible cause [16]. Alternatively, a pre-existing extension deficit caused by osteoarthritis could also exacerbate the postoperative tension on the osteosynthesis material, leading to early material dislocation or insufficiency. In their systematic review, Matthews et al. found that older age (> 65) and a multi-fragment fracture pattern were predictive factors for material failure and limited postoperative mobility [16].

In our cohort, patients who experienced *atraumatic fragment dislocation* (the most common complication) were significantly older than those with other complication types. The reasons for this type of complication might be manifold with 70% of fractures being comminuted and 50% involving the distal pole. However, only 20% of patients received a preoperative CT scan. Therefore, in addition to the potential cause of fixation failure due to the comminuted fracture pattern in combination with impaired bone quality, one possible explanation for the occurrence of an *atraumatic fragment dislocation* could be the discrepancy between the severity of the comminuted fracture and the infrequence or absence of a CT scan. In general, a conventional x-ray underestimates the extent of the fracture and the number of fragments [21]. Therefore, an inadequate understanding of the extent of the fracture and the fragments themselves, especially in the presence of concomitant distal pole fractures, may possibly increase the risk of *atraumatic fragment dislocation*. Lazaro et al. have emphasized the significance of a preoperative CT scan in demonstrating the distal pole’s involvement in 88% of cases, frequently in a comminuted manner, but which is only detectable in 44% of cases through plain radiographs [21]. Thus, CT scans resulted in a modification of the treatment plan in almost half of the cases. Therefore, it is highly recommended to routinely perform preoperative CT scans to select a surgical treatment adapted to the fracture and to lower the risk of atraumatic fragment dislocation. Our findings revealed that AO/OTA classification had a significant impact on the occurrence of complications. Specifically, type-C fractures were the only ones in this cohort to exhibit complications and the frequency of complications increased with the complexity of the fracture. This becomes especially apparent when complex comminuted fractures are not identified as such (due to the absence of CT scanning) or are inadequately stabilized (due to insufficient osteosynthesis).

Furthermore, the most common complication of *atraumatic fragment dislocation* in this cohort occurred early, after 4–5 weeks. Notably, the risk of secondary dislocation was increased during the initial postoperative phase, when there was still restricted range of knee motion and partial weight bearing, underscoring the crucial need for a stable fixation with an osteosynthesis method that guarantees primary stability of all relevant fragments before complete bone healing. In this context, it was not surprising that *material insufficiency/dislocation* was the second most common complication, observed exclusively after TBW. Consistent with current literature, our data highlights the inability of TBW to provide the necessary primary stability, resulting in a comparably high risk of material insufficiency [3, 15, 22]. Therefore, it must be questioned if the classic tension band wiring is still the appropriate surgical treatment for comminuted fractures. While TBW is the standard treatment recommended by the AO [23], it remains the most commonly used surgical procedure and preferred treatment in clinical practice for both simple transverse fractures (approximately 69%) and comminuted fractures (approximately 57%) in German-speaking countries [15]. The significantly higher material cost of LPO compared to TBW may be one reason. However, any revision surgery in the event of a failed primary TBW will exceed this cost. Moreover, there is no biomechanical evidence that TBW is effective in converting tension forces into compression forces on the patella [24]. In 1997, Labitzke et al. reported that the eccentric placement of a rigid cerclage results in inadequate compression, particularly on the articular side of the fracture region [25]. Comminuted fractures often result in residual articular incongruity, which can lead to post-traumatic retropatellar osteoarthritis in up to 70% of cases [26]. The functional results after TBW are often unsatisfactory and exhibit functional limitations in 30–70% of patients [3, 27]. This is in part probably due to the comparatively high rates of postoperative complications of 22–56% [3, 6, 11, 28]. In the literature, the most common types of complications of TBW involve secondary fragment dislocation, as well as material loosing and dislocation, which is consistent with the results of our study.

While TBW did not significantly increase overall rates of complications, certain complications, particularly implant-related issues such as *atraumatic fragment dislocation* and *material insufficiency/dislocation* (accounting for 50% of complications), were found to be TBW-related. However, it must be taken into account that the number of complex fracture patterns was comparatively higher in the TBW group and could therefore also influence the occurrence of complications in this subgroup.

Functional knee scores were generally lower and pain levels were significantly higher following complications, especially after complications such as *limited range of motion* or *traumatic refracture*. These results persisted for up to 5 years. The functional scores of patients without complications were comparable to those reported in the current literature. However, tension band wiring generally resulted in worse score values [8, 29, 30]. These persistently poorer results following complications emphasize the critical importance of avoiding initial complications by all means. Patients who experienced an implant-related complication of *atraumatic fragment dislocation* or *material insufficiency* displayed significant improved functional scores up to 5 years in comparison to other types of complications. Further, these patients did not exhibit significant functional differences when compared to those who reported no complications. One possible explanation is that almost every patient who experienced an *atraumatic fragment dislocation* and *material insufficiency/dislocation* received surgical revision, potentially leading to a more secure and adequate osteosynthesis and resulting in better functional outcomes in the intermediate to long term. In this context, Müller and Frosch demonstrated that early revision surgery after failure of primary osteosynthesis of patella fractures in combination with secondary anatomic reconstruction and good radiological results lead to satisfactory functional outcomes [10]. These findings are consistent with the good functional outcomes achieved after a complication of *atraumatic fragment dislocation* in this cohort. This may be due to the fact that all patients underwent early revision surgery with re-osteosynthesis.

The study has certain limitations. The use of TBW for surgical treatment was quite high, accounting for approximately two-thirds of all cases. Rau et al. analyzed the epidemiological and surgical treatment data of patella fractures in Germany from 2006 to 2020, and their results showed that although there was a rapid and consistent increase in the use of locking plates, traditional TBW was the most commonly used procedure during our study period (2013–2018), especially in the initial phase [17]. Additionally, the study by Fehske et al. [15] showed that approximately 57% of comminuted fractures were treated with TBW, which might help to explain why TBW was also used more frequently than LPO in this cohort, resulting in an uneven distribution. The functional evaluation at the final follow-up based on the subjective assessment of the study participants as neither clinical nor radiological examination was performed. This limits the evaluation and correlation of symptoms as pain or functional restrictions to comparably worse functional scores. Furthermore, osteoporosis was not routinely assessed by DXA in all patients, but only through the anamnesis and by patient reports. This could potentially lead to an underestimation of the prevalence of osteoporosis. Finally, the study is subject to selection bias because data were not collected from all patients who underwent surgery at all centers during the study period due to incomplete questionnaires, non-response, or refusal to participate.

## Conclusion

Around 150 years after the first surgery for a patella fracture under aseptic conditions and despite the most modern diagnostics and various osteosynthesis procedures, this injury still remains a therapeutic challenge with in part severe and high rates of complications. TBW is associated with comparably high implant-related complications. A comprehensive understanding of the fracture morphology and tailored osteosynthesis are crucial to reduce the risk of complications and achieve an optimal functional outcome. Preoperative CT scans are still too infrequently performed, even though they can help to select a surgical treatment appropriate for the fracture and reduce the risk of atraumatic fragment dislocation, especially in comminuted fractures. Biomechanical and clinical studies provide insight into postoperative function and rates of complications for different osteosynthesis methods, but current clinical practice still may differ from these evidence-based results.
